# Rubella Immunity among Pregnant Women in Jeddah, Western Region of Saudi Arabia

**DOI:** 10.1155/2014/659838

**Published:** 2014-06-19

**Authors:** Sharifa A. Alsibiani

**Affiliations:** Department of Obstetrics and Gynecology, Faculty of Medicine, King Abdulaziz University, P.O. Box 122415, Jeddah 21332, Saudi Arabia

## Abstract

To determine the presence of rubella immunity among pregnant women attending their first prenatal visit in Jeddah, Saudi Arabia, a retrospective, descriptive, cross-sectional, hospital-based study (prevalence study) was undertaken. A total of 10276 women attending prenatal clinics between January 1, 2008, and December 31, 2011 were included. Rubella screening tests (immunoglobulins: IgG and IgM), rubella antibody titer levels, patient age, gravidity, parity, and the number of previous abortions were analyzed. No patients tested IgM positive, and 9410 (91.6%) were immune (IgG positive); the remaining 866 (8.4%) were susceptible. There were no significant differences in gravidity, parity, or the number of previous abortions between immune and nonimmune groups. In contrast, the immunity rate decreased with increasing age, with a significant difference between the youngest age group (15–19 years) and the oldest age group (40–49 years) (*P* = 0.0005; odds ratio, 2.86; 95% confidence interval, 1.7–4.7). Rubella immunity among pregnant women was high (91.6%) but decreased significantly with increasing age. A possible explanation for this is the change in the rubella vaccination policy in Saudi Arabia in 2002, from 1 dose to 2 doses. In addition, antibody levels begin to decline after vaccination and natural infection.

## 1. Introduction

Rubella is a mild, self-limiting, viral infection that causes illness worldwide. It is caused by a non-arthropod-borne member of the family Togaviridae [1]. At least half of all primary rubella infections are undiagnosed because of the subclinical nature of the infection. Although the virus causes only a mild infection in healthy adults, an infection in a pregnant woman can be devastating to the fetus [2]. If a rubella virus infection occurs early during pregnancy, there is a 90% chance of passing the virus on to the fetus. A maternal rubella infection during the first trimester is associated with an increased risk of intrauterine death, spontaneous abortion, and congenital malformations known as congenital rubella syndrome (CRS), which affects all organs in the developing fetus [2]. CRS also has late-onset manifestations, including autism, diabetes mellitus, and thyroiditis [3]. According to the World Health Organization (WHO) estimates, >100,000 children are born annually with CRS worldwide [3]. Unfortunately, there are no recent reports on the incidence rate of CRS in Saudi Arabia; however, some studies conducted in the 1980s and 1990s reported that the incidence rates of CRS per 100000 live births were 27 and 7, respectively [4].

There is no specific therapy for maternal or congenital rubella infection. The value of immunoglobulin administered after viral exposure early in pregnancy has not yet been established. Thus, the primary means of preventing CRS is rubella immunization. The live-attenuated rubella vaccine has been available for use since 1969. It is highly effective; a single dose of the most commonly used RA27/3 rubella vaccine strain leads to seroconversion in at least 95% of vaccines and is thought to afford lifelong protection [2]. Many developed countries have been able to utilize the vaccine effectively, reducing the prevalence of rubella and preventing the consequences of CRS [5].

In Saudi Arabia in 1978, the initial selective rubella vaccination policy was targeted towards prepubescent schoolgirls (11–14 years) in order to protect their future pregnancies. In 1982, the “1402H” vaccination against rubella as part of the measles and mumps vaccine (MMR) was licensed, and a combined vaccination policy was adopted. The vaccine is offered to all children of both sexes at 12 months and to prepubescent schoolgirls (11–14 years) [6]. Since the introduction of the first uniform expanded program of immunization (EPI) in Saudi Arabia in 1991, the rubella vaccine has been given as part of the MMR vaccination. Its schedule has been changed several times by modifications in the EPI schedule (Table 1) aiming to ensure high immunity and coverage [7, 8]. Since 1995, Arab Gulf countries, including Bahrain, Kuwait, Oman, Qatar, Saudi Arabia, and the United Arab Emirates, have also given special attention to the control of rubella [3].

Saudi Arabia implements postpartum vaccination for rubella seronegative mothers. Antenatal rubella IgG antibody screening is routinely performed during the first antenatal visit, enabling the identification of susceptible women, who can subsequently receive postpartum vaccination. However, because of the expense of screening, it is not recommended in all countries [2]. Inoculation with the rubella vaccine should be avoided during pregnancy because of the theoretical, but unproven, teratogenic risk [2]. If a pregnancy is being planned, then an interval of one month should be observed after a rubella vaccination [2].

Since most Saudi women of childbearing age are included in the vaccination program, determining the status of rubella immunity among women of childbearing age is highly important to evaluate the current vaccination strategies and decide whether additional modifications to the EPI are required. There were two primary goals of the current study: first, to determine the prevalence of rubella immunity among pregnant women attending their first antenatal visit in two hospitals types in Jeddah, in the Western region of Saudi Arabia, and second, to identify potential predictors of rubella immunity including age, gravidity, parity, or the number of previous abortions.

## 2. Materials and Methods 

This is a retrospective, descriptive, cross-sectional, hospital-based study (prevalence study). Data of routine rubella screenings of women attending prenatal clinics were reviewed in both a teaching hospital and two private clinics in private hospitals located in Jeddah, Saudi Arabia. King Abdulaziz University Hospital is the main teaching hospital of the Western region and serves a mixture of social classes. Both hospital types provide tertiary medical care for the population of the Western region. The study included 10276 women who received prenatal care over a 4-year period from January 1, 2008, to December 31, 2011.

The study utilized the following measures and/or variables: rubella screening tests (IgG and IgM) (presence of rubella: yes or no), rubella antibody titer levels (≥10 IU/mL = positive; ≤4.99 IU/mL = negative; 5–9.99 IU/mL = equivocal), patient age (15–49 years of age), gravidity (number of pregnancies), parity (number of deliveries), and the number of abortions (before 20 weeks of gestation or weighing < 500 grams).

The patient data were stratified by age (≤19 years, 20–29 years, 30–39 years, and 40–49 years), gravidity (primigravida [G1], multigravida [G2–G5], and grand-multigravida [>G5]), parity (nulliparous [P0], para 1 [P1], and multiparous [>P1]), and history of previous abortions (with or without a history of abortion).

Rubella IgG antibodies were quantified using an enzyme-linked immunosorbent assay (ELISA) BPiii (Dade Behring, Germany). Although 4 IU/mL is used as cutoff in commercial ELISAs, patients were considered positive for rubella based upon guidelines provided by the WHO, [1] as described above. Approval for the study was granted by the Research Ethics Committee of King Abdulaziz University Hospital.

Statistical analysis was performed using the Statistical Package for the Social Science (SPSS), version 16 for Windows. Continuous variables were summarized using descriptive statistics in terms of means ± standard deviations and 95% confidence intervals (95% CI). The prevalence among different groups was compared using the chi-square test for categorical variables; a* t*-test was used to measure the difference in means between two groups. A *P* value < 0.05 was considered statistically significant. Logistic regression analysis was performed to assess predictors of rubella susceptibility.

## 3. Results

During the study period, 10276 women received prenatal care. Mean patient characteristics including the factors analyzed as predictors of immunity are shown in Table 2. Patient age ranged from 15 to 48 years; gravidity ranged from 1 to 17; mean parity ranged from 0 to 14; and the number of abortions within the medical history ranged from 0 to 11.

The first research question addressed the prevalence of rubella antibodies in the group of women. Of the 10276 women that were screened for rubella immunity (IgG and IgM), the majority (91.6%) were immune or IgG positive (+ve) and the remainder (8.4%) were considered susceptible to rubella (IgG negative [−ve]). No patients tested positive for rubella (IgM −ve).

Immunity to rubella was queried as a function of several patient factors, including gravidity, parity, and history of abortion. Table 2 shows that there were no statistically significant differences between the immune and nonimmune (susceptible) cases with regard to gravidity, parity, or abortion.

The second aim investigated factors affecting susceptibility between immune and nonimmune pregnant women. The analyses indicated that there were no statistically significant differences between the two groups in terms of gravidity, parity, or the number of previous abortions, whereas age was significantly different between the two groups (*P* < 0.001) (Table 2). The immunity rate decreased significantly with age (*P* = 0.0005) and was the highest in the 15- to 19-year-old group and the lowest in women 40 years old (Table 3).

Using the 40- to 49-year-old age group as the reference category, logistic regression analysis revealed that the youngest age group (15–19 years) had better immunity compared with the oldest age group. In contrast, the immunity rate in the other two age groups did not differ significantly from the oldest age group (Table 4).

## 4. Discussion

To prevent CRS, primary strategies have included both rubella vaccination of the general population and screening for rubella in pregnant women. Many countries have conducted serosurveys to determine the proportion of women of childbearing age who are susceptible to rubella (negative for rubella-specific IgG). Variable rates of rubella immunity among pregnant women or women of childbearing age have been observed in different regions of the world. As reported by Jarour et al. [9], in developing countries, immunity rates are as low as 32%, or as high as 95.3% in Mozambique, and up to 98% in South Africa [10, 11]. On the other hand, the rates of immunity to rubella are higher in Western European countries, such as Finland and the Netherlands, with susceptibility rates as low as <5%, [12] even though levels of immunity widely differ in the general population and specific age groups [9]. In a review of serosurveys on CRS and rubella susceptibility among women of childbearing age in 45 developing countries prior to the introduction of the rubella vaccine, the WHO classified some Arabian countries, including Jordan, Libya, Morocco, and Tunisia, within the top 13 developing countries to have protection levels of >90% against rubella among women of childbearing age [3].

Termination of pregnancy for CRS is prohibited in Saudi Arabia. Thus, immunization against rubella is very important because it is the only way of preventing CRS. Two principal strategies may be considered: (1) selective vaccination of adolescent and adult females and (2) routine vaccination of all young children. The first strategy may prevent CRS but does not control rubella. The second strategy can potentially control and eventually eliminate rubella and CRS but carries the risk of increasing the average age of patients with rubella infection. A vaccination program would decrease circulation of the virus in the community; however, in women not included on the vaccination program or if vaccination coverage is insufficient, the decrease in viral prevalence will reduce the chance of developing immunity by natural infection. Accordingly, this could carry the risk of increasing the average age of women who develop a rubella infection, resulting in an increased number of CRS cases [13]. The implementation of either strategy is dependent on many issues, such as infrastructure, goals of the program, and funding [1]. According to Plotkin, vaccination of all infants will probably eradicate CRS in 30–40 years, vaccination of all schoolgirls will presumably eradicate CRS in 10–20 years, and vaccination of adult women will eradicate CRS immediately, but only if 100% are immunized [14].

Twenty-five years (until the time of data collection for this study) after the MMR vaccine was licensed and approximately 16 years after the first uniform EPI was released in Saudi Arabia, the overall seropositivity rate in this study among pregnant women (91.6%) was similar to previous rubella seroprevalence studies conducted in the 1980s and 1990s [6, 15–20], before or shortly after the immunization program was adopted, respectively. The current results are also similar to those for Jordan (90.9%), Kuwait (92.3%), Oman (92%), Iran (94.6%), the United States (91%), and Canada (91.8%), and seropositivity in the current research is greater than the percentages in Sri Lanka (76%) and Nigeria (68.5%) [9, 21–24].

De Paschale et al. [25] stressed an important point when comparing published data. It is important to consider the method used to determine the presence of antibodies and the antibody titer, as well as the guidelines used as an index of positivity. It has been suggested that immunity is provided by serum IgG levels of 10–15 IU/mL [25]. However, in a study conducted 20 years after vaccination with two doses, all of the subjects with IgG levels >4 IU/mL were considered to be immune; however, 17% had levels <10 IU/mL and 36% had levels <15 IU/mL. Here, because the fetuses of women with lower antibody values are at risk of developing CRS, we defined positivity as levels ≥10 IU/mL. Consequently, the fact that 8.7% of these Saudi women were seronegative may have been because of absence of a vaccination program, an incomplete antibody response, or a decline in antibodies over time.

Unlike the current study in Saudi women, in Kuwait and Argentina, rubella seroprevalence has been associated with parity [21, 26]. In addition, lower rubella susceptibility rates in parous women have been documented in England [27].

Importantly, this study found that the immunity rate among women decreased significantly and progressively with increasing age. These data suggest that older Saudi women have a potentially increased risk for CRS. Worldwide seroepidemiological studies have indicated that the acquisition of immunity to rubella is related to both age and social class [21]. However, the current finding is in contrast to those of some local studies conducted in the 1990s [6, 15–20]. Those studies showed a direct relationship between rubella IgG prevalence and age in a healthy population. A study conducted in Kuwait in 1995 showed that women aged 30–34 were more likely to be nonimmune compared with females <20 years old and those >35 years old [21]. In Jordan [9], the immunity rate was significantly lower in women aged 15–19 (83%) compared with women aged 40–49 (92.2%). A study in Indian women also revealed that seropositivity in pregnant women increased with age [24].

The decrease in rubella IgG prevalence among women as a function of age, despite the introduction of rubella vaccination in infants, could be explained by a paradoxical effect. In a population, vaccination of infants reduces immunity in nonimmunized women by reducing natural exposure to the rubella virus because its circulation in the population is reduced. This increases the susceptibility of these adults to residual exposures during pregnancies. However, this issue is temporary because the previously immunized infants grow to become adults, while the nonimmunized adults can now be immunized by a single mass campaign involving vaccination of women (or both sexes) up to 39 years of age. Catch-up vaccination in children up to 15 years of age may also greatly reduce the risk of this paradoxical effect [28]. A study conducted in 2004, in Jeddah preschool- and school-aged students, showed a high prevalence of rubella IgG (90%), which was higher among vaccinated versus nonvaccinated students and decreased with age. In nonvaccinated children, the percentage of seropositivity increased with age. The author explained this effect by suggesting that there is variability in the maintenance of antibodies after rubella vaccination [29].

Other explanations for the decrease in rubella IgG prevalence with age in this study are possible. Saudi Arabia health regulations implemented changes in the dosage of the rubella in the immunization program. Prior to 2002, mostly, only a single dose was administered. Since that time, the number of doses was increased to two. Although the difference is slight, one dose is less efficacious than two [25]. Furthermore, observations made today are the result of vaccinations given years ago, and the antibodies may have been lost with time. Antibody levels decline over time after vaccination and after natural infection; a minimal annual antibody decay rate of −2.9% has been reported in the literature [25].

On the other hand, several factors should be considered when examining the importance of these findings among the current population. First, although most of the childbearing Saudi population is young, the social desire for large families requires that older women continue to bear children, and their loss of immunity with age could put them at risk of rubella infection and increase the likelihood of CRS. Second, the immunity within the 20- to 29-year age group and the 30- to 39-year age group did not differ significantly from the oldest age group. Third, a small percentage of women (approximately 1%) may not respond to the rubella vaccine by forming IgG or IgM antibodies. Thus, seronegative women of childbearing age who are vaccinated should be tested for seroconversion at least 8 weeks after their last vaccination. Finally, IgG levels between 10 and 20 IU/mL are considered low. Reinfection is possible in women with these levels, and the infection is mostly asymptomatic, posing a potential risk to the fetus. The incidence of CRS after maternal reinfection during the first trimester has been estimated to be 8%; however, CRS has not been reported after maternal reinfection beyond 12 weeks of gestation [30]. Therefore, a booster dose of rubella vaccine is recommended particularly in women anticipating pregnancy or in those who have just given birth and are planning to have more pregnancies [1].

Because of the important findings of this study that showed a decrease in rubella immunity with increase in age in Saudi women, there are three recommendations. First, a prenatal screening for rubella should be conducted irrespective of a history of previous vaccination, clinical rubella, or previous positive rubella antibody status. Second, postnatal vaccination should be implemented for all seronegative women whose IgG levels are <20 IU/mL. Finally, rubella screening tests for premarital and preuniversity entry individuals will help to achieve catch-up vaccination for susceptible females, thereby decreasing circulation of the virus.

## 5. Conclusions

In pregnant Saudi women, rubella immunity is high (91.6%) but decreases significantly with increasing age. The percentage of nonimmune pregnant women has not decreased in the Saudi pregnant population since 1980s, despite the introduction of an immunization program >20 years ago, suggesting that any rubella vaccination programs need to be continuously evaluated to ensure that the intervention reaches the specified goals. Therefore, to achieve catch-up vaccinations for susceptible females, the current study advocates a continuation of the vaccination program among infants, antenatal screening with postnatal vaccination for nonimmunized women and those with low rubella IgG (<20 IU/mL), and the addition of rubella screening to premarital and pre-university entry tests. Finally, the WHO identified rubella as one of the neglected diseases. Thus, the results of the present study may be beneficial globally and may not be solely limited to issues related to the prevalence in Saudi Arabia.

## Figures and Tables

**Table 1:** Summary of the history of the implemented rubella vaccination programs in Saudi Arabia.

Year	Dose	Age and sex	Vaccine strain
1978	1	School girls (11–14 years)	Wistar RA 27/3
1982	2	Boys and girls (12 months)	MMR
		School girls (11–14 years)	
1991∗	1	Boys and girls (12 months)	MMR
2002	2	Boys and girls (12 and 24 months)	MMR
2009	3	Boys and girls (12 and 24 months and 4–6 years)	MMR
2013	3	Boys and girls (12 and 24 months and age of school entry [~7 years])	MMR

*In 1991 the first uniform expanded program of immunization (EPI) was initiated in Saudi Arabia.

MMR: measles, mumps, rubella.

**Table 2:** Results comparing the immune and nonimmune pregnant women.

	Rubella-immune IgG +ve	Rubella-susceptible IgG −ve	Total	*t*-test, *P* value
*N* (%)	9410 (91.6%)	866 (8.4%)	10276 (100%)	
Age (years)	27.60 ± 6.1	31.3 ± 6.1	27.7 ± 6.1	3.291∗, 0.001
Gravidity	3.5 ± 2.5	3.39 ± 2.6	3.5 ± 2.5	0.944∗, 0.185
Parity	2.1 ± 2.2	1.99 ± 2.2	2.1 ± 2.2	0.755∗, 0.331
Abortion	0.47 ± 0.95	0.43 ± 0.89	0.479 ± 0.97	0.515∗, 0.276

All data are presented as means ± standard deviation. *P* = probability value considered significant at *P* < 0.05. *t-test for the difference between the two means.

IgG +ve: immunoglobulin G positive; IgG −ve: immunoglobulin G negative.

**Table 3:** Immunity rate to rubella as a function of age in pregnant women from Jeddah and the Western region of Saudi Arabia.

Age (years)	Total women tested	Immune women		
		Number	% (95% CI)	*χ* ^2^ (*P*)
15–19	703	674	95.9% (94.4–97.3%)	
20–29	5880	5386	91.6% (90.9–92.3%)	21.952 (0.0005)
30–39	3294	2994	90.9% (89.9–91.8%)	
40–49	374	333	89.0% (85.9–92.2%)	

Total	10251∗	9387	91.6% (91.0–92.1%)	

95% CI: 95% confidence interval; *χ* ^2^: chi-square test.

∗Data from 25 patients were unavailable.

**Table 4:** Logistic regression analysis of age groups on the likelihood of the presence of rubella antibodies.

Age (years)	*P*	Odds ratio (95% CI)
15–19	0.0005	2.9 (1.7–4.7)
20–29	0.087	1.34 (0.96–1.9)
30–39	0.733	0.94 (0.66–1.3)
40–49 (reference)	—	1

95% CI: 95% confidence interval.
